# Supplementary material for: Atlantic sediments reveal interacting environmental and physiological controls on coccolithophore calcite production
Source: Nat Commun. 2026 May 28;17:4722. doi: 10.1038/s41467-026-73162-5 (PMC13219798; doi:10.1038/s41467-026-73162-5)
Supplement: Supplementary file 1 — Supplementary Information [file 41467_2026_73162_MOESM1_ESM.pdf]

Supplementary Information for:

Atlantic sediments reveal interacting environmental and physiological controls on coccolithophore calcite production

Alba González-Lanchas [1*], Karl-Heinz Baumann [2], Heather M. Stoll [3], José-Abel Flores [4], Miguel A. Fuertes [5] and Rosalind E.M. Rickaby [1]

[1]Department of Earth Sciences, University of Oxford, Oxford, United Kingdom. [2]Department of Geosciences, University of Bremen, Bremen, Germany. [3]Department of Earth and Planetary Sciences, ETH Zürich, Zürich, Switzerland. [4]Department of Geology, University of Salamanca, Salamanca, Spain. [5]Department of Didactics of Mathematics and Experimental Sciences, University of Salamanca, Salamanca, Spain.

[*]email: alba.gonzalez-lanchas@earth.ox.ac.uk

Contents of this file

Supplementary Figures **1** to **11**
Supplementary Tables **1** to **5**
Supplementary Methods **1** to **3**
Supplementary Notes **1** to **4**

Supplementary Figures

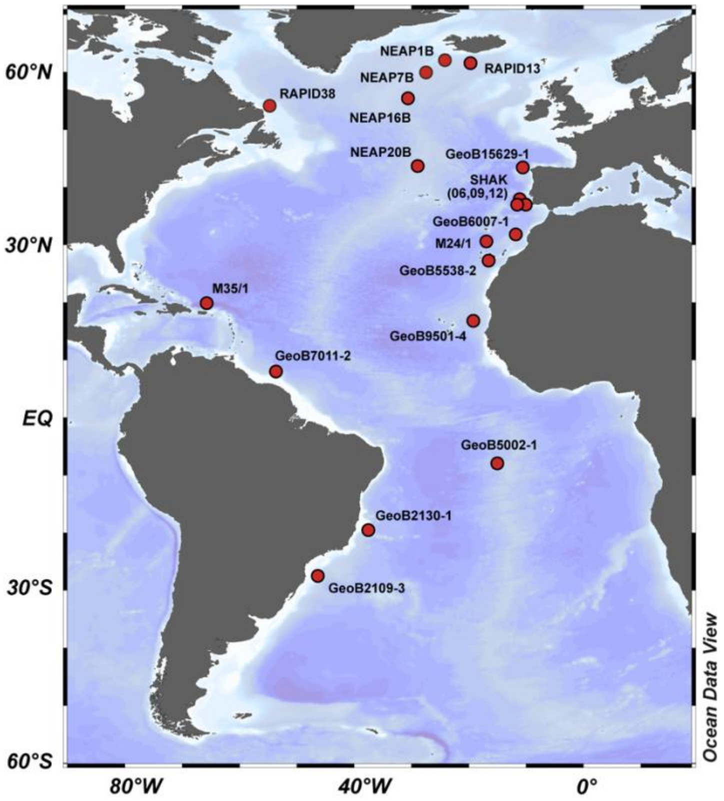

Supplementary Figure 1. Spatial distribution of surface sediment samples (red dots) overlaid on a map generated using Ocean Data View [1]. The bathymetry from ETOPO5 [2] is incorporated by ODV for the map representation. See the list with complete sample reference name in the Supplementary Table 1.

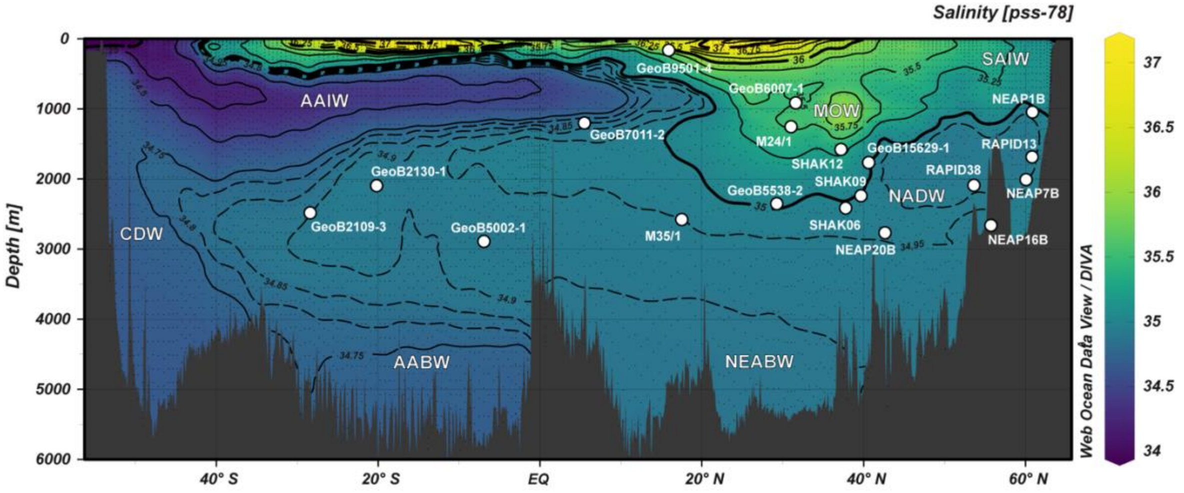

Supplementary Figure 2 (Figure S2). Vertical distribution of surface sediment samples (white dots) represented over a map of salinity generated using the webODV Explore service [3]. The salinity data correspond to the A16 Atlantic transect from the World Ocean Circulation Experiment (WOCE) [4]. Abbreviations indicate water masses: CDW: Circumpolar Deep Water; AAIW: Antarctic Intermediate Water; AABW: Antarctic Bottom Water; NEABW: Northeast Atlantic Bottom Water; MOW: Mediterranean Outflow Water; NADW: North Atlantic Deep Water and SAIW: Subarctic Intermediate Water. See the list with complete sample reference name in the Supplementary Table 1.

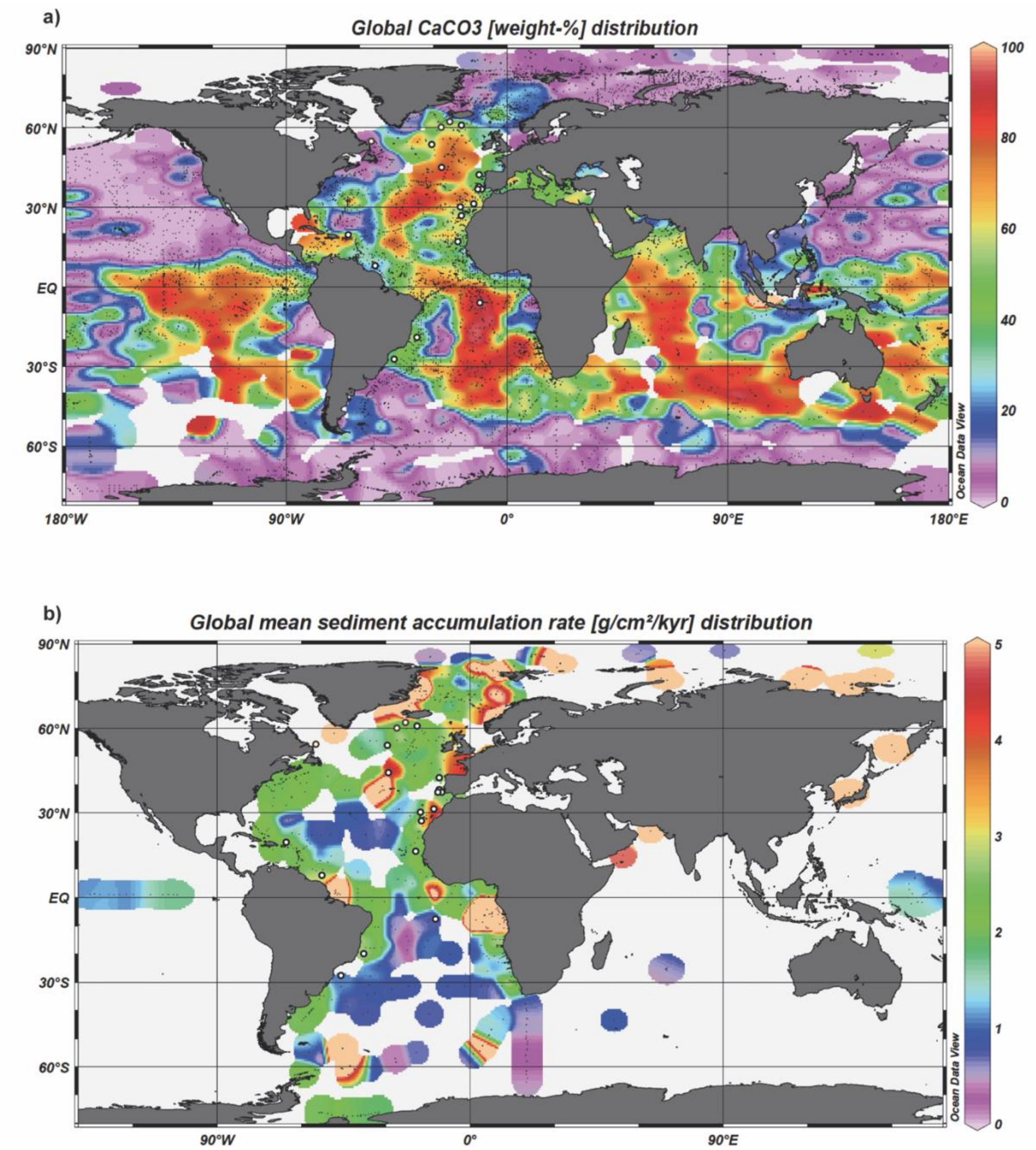

Supplementary Figure 3. Representation of surface sediment samples (white dots) overlaid maps showing **a)** the distribution of global solid surface sediment calcium carbonate data ($CaCO_3$; %); and **b)** the estimated global solid surface sediment mean accumulation rates (g cm^2 kyr^{-1}). The maps in were sourced from the Integrated Data Sets of the EU FP5 Research Project ORFOIS database [5]. All ORFOIS data related to global solid surface sediment calcium carbonate %, in a) and global solid surface sediment mean accumulation rate estimates, in b) corresponds to the citation set compiled in: http://orfois.wdc-mare.org/AR_References.php and http://orfois.wdc-mare.org/CaCO3_References.php, respectively.

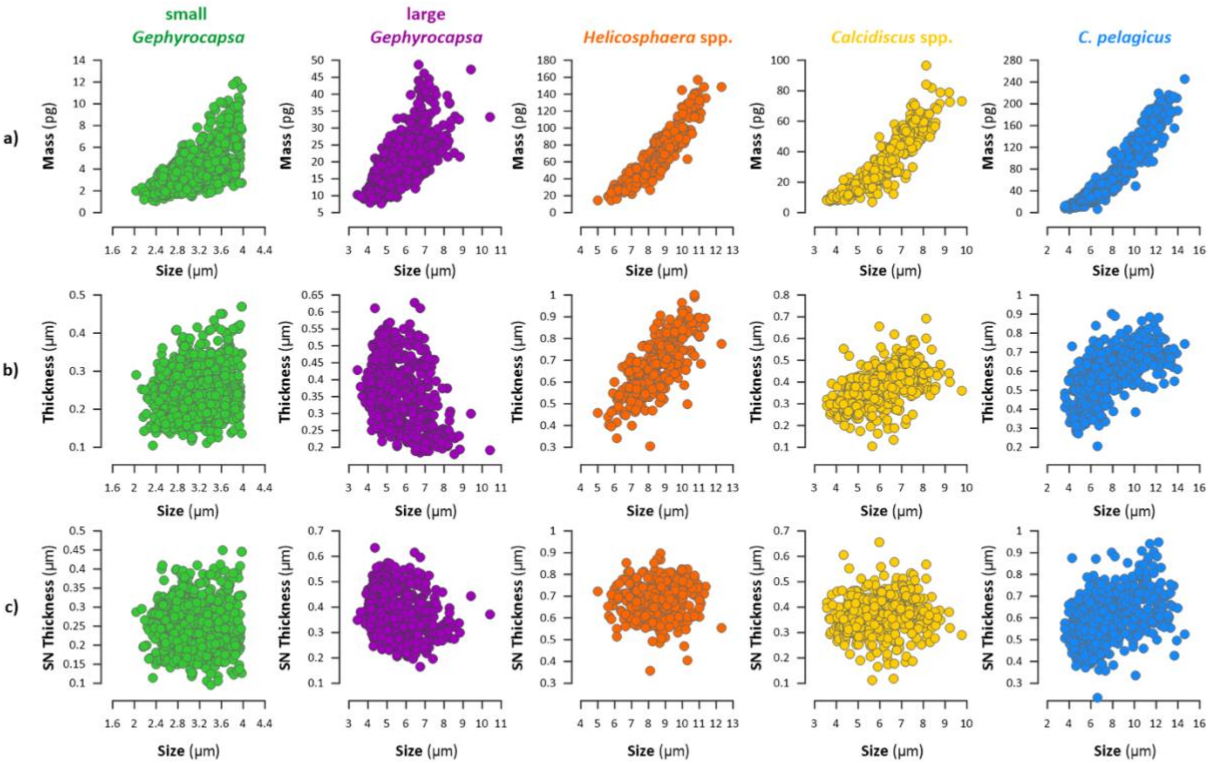

Supplementary Figure 4. Cross correlation relationship between coccolith morphometric parameters **a)** Size (µm) and mass (pg) **b)** Size (µm) and Thickness (µm) and **c)** Size (µm) and Size Normalized (SN) Thickness (µm). Taxa are color-coded as follows: small *Gephyrocapsa* (dark green; n = 920); large *Gephyrocapsa* (purple; n = 516); *Helicosphaera* spp. (orange; n = 334); *Calcidiscus* spp. (yellow; n = 454) and *C. pelagicus* (blue; n = 511).

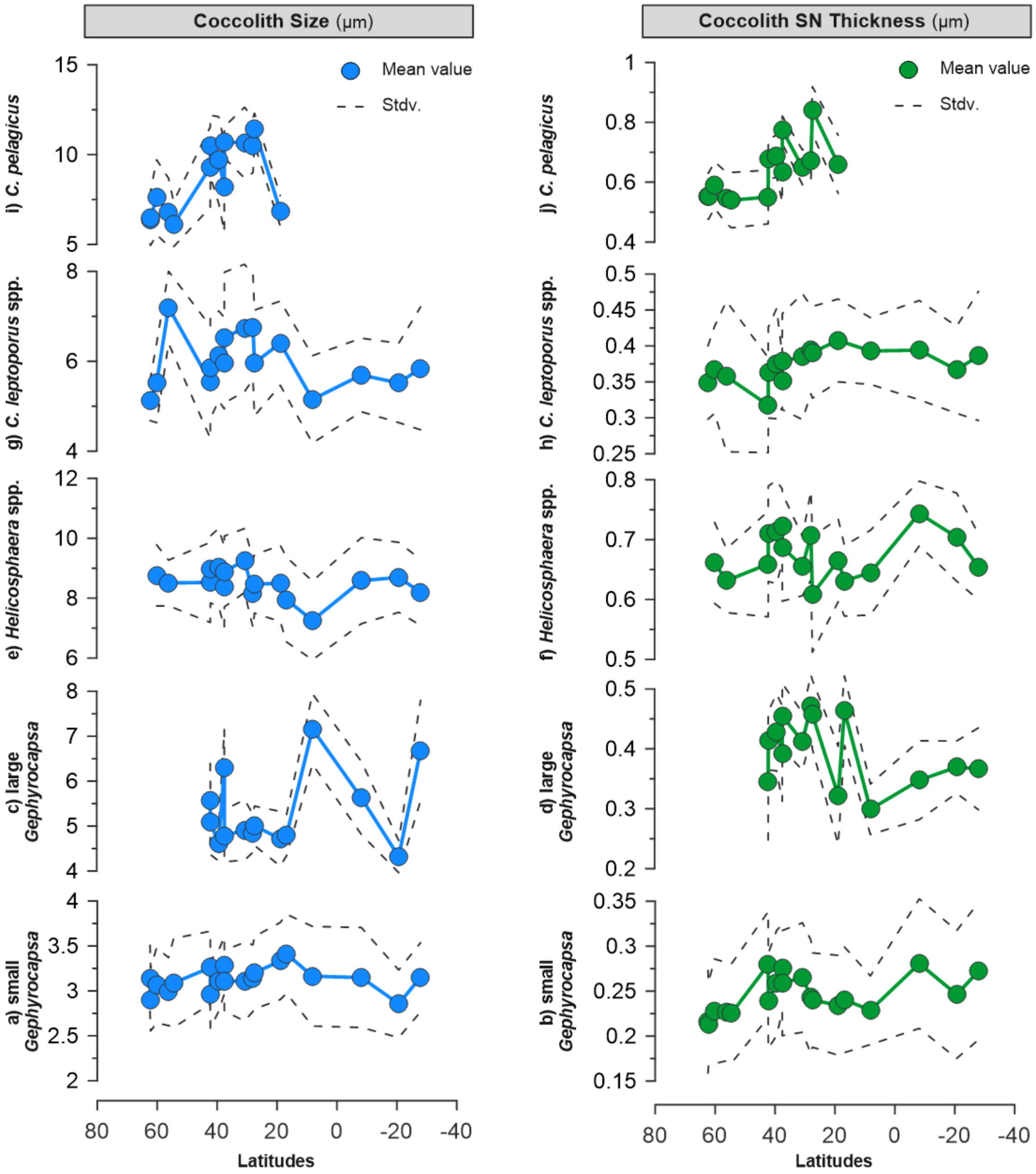

Supplementary Figure 5. Latitudinal variability in profiles of Size (µm) and Size Normalized (SN) Thickness (µm) for the coccolithophore taxa analyzed in this study. **a)** and **b)** small *Gephyrocapsa*; **c)** and **d)** large *Gephyrocapsa*; **e)** and **f)** *Helicosphaera* spp.; **g)** and **h)** *Calcidiscus* spp.; **i)** and **j)** *C. pelagicus*. Dots represent average values at each sampling point, and dashed lines indicate standard deviation (stdv.) values.

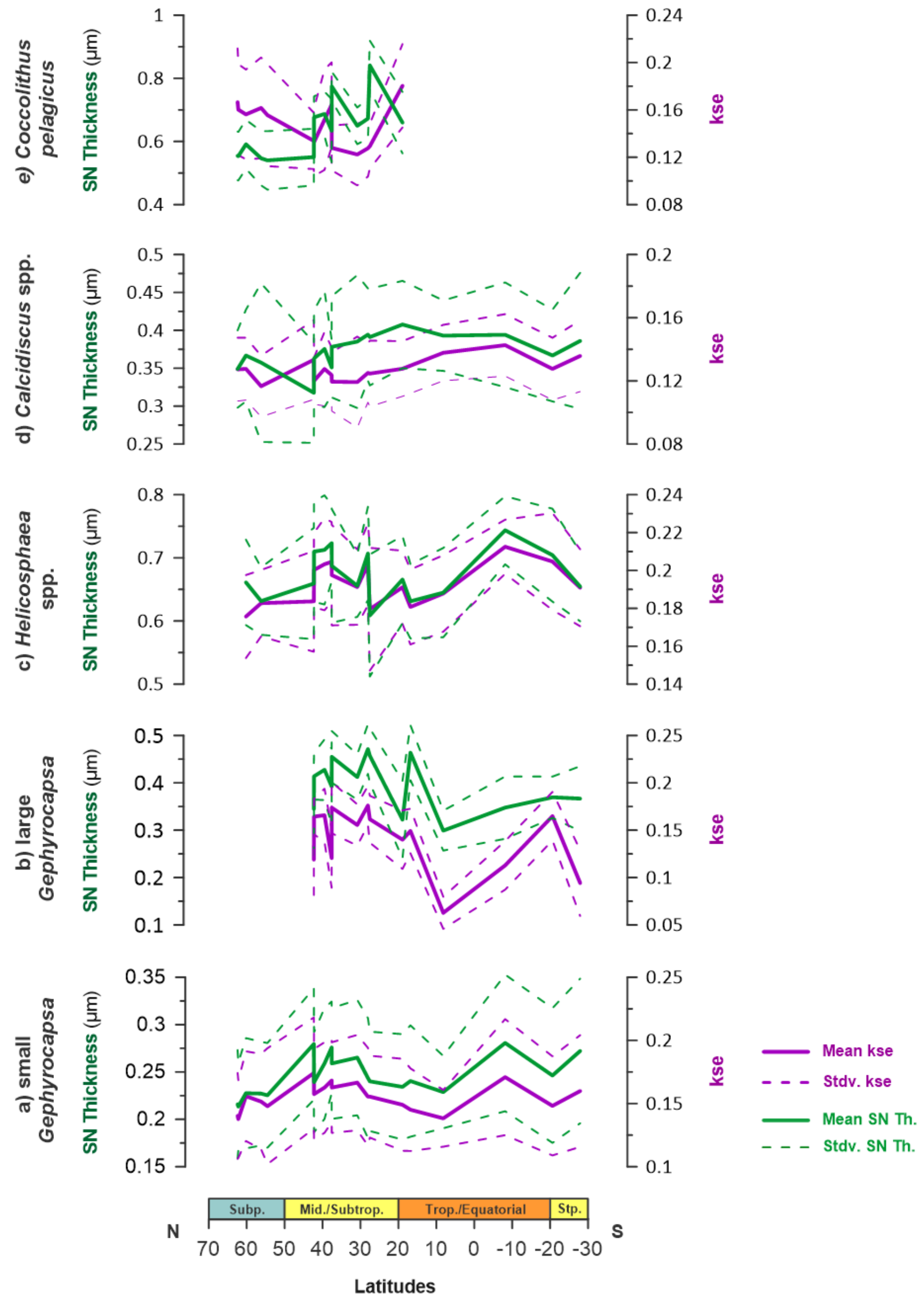

Supplementary Figure 6. Latitudinal variability in profiles of Size Normalized (SN) Thickness (µm) and elliptical shape factor (kse; dimensionless) for the coccolithophore taxa analyzed in this study. **a)** small *Gephyrocapsa*; **b)** large *Gephyrocapsa*; **c)** *Helicosphaera* spp.; **d)** *Calcidiscus* spp. and **e)** *C. pelagicus*. Solid lines connect average values at each sampling point, and dashed lines represent standard deviation (stdv.) values.

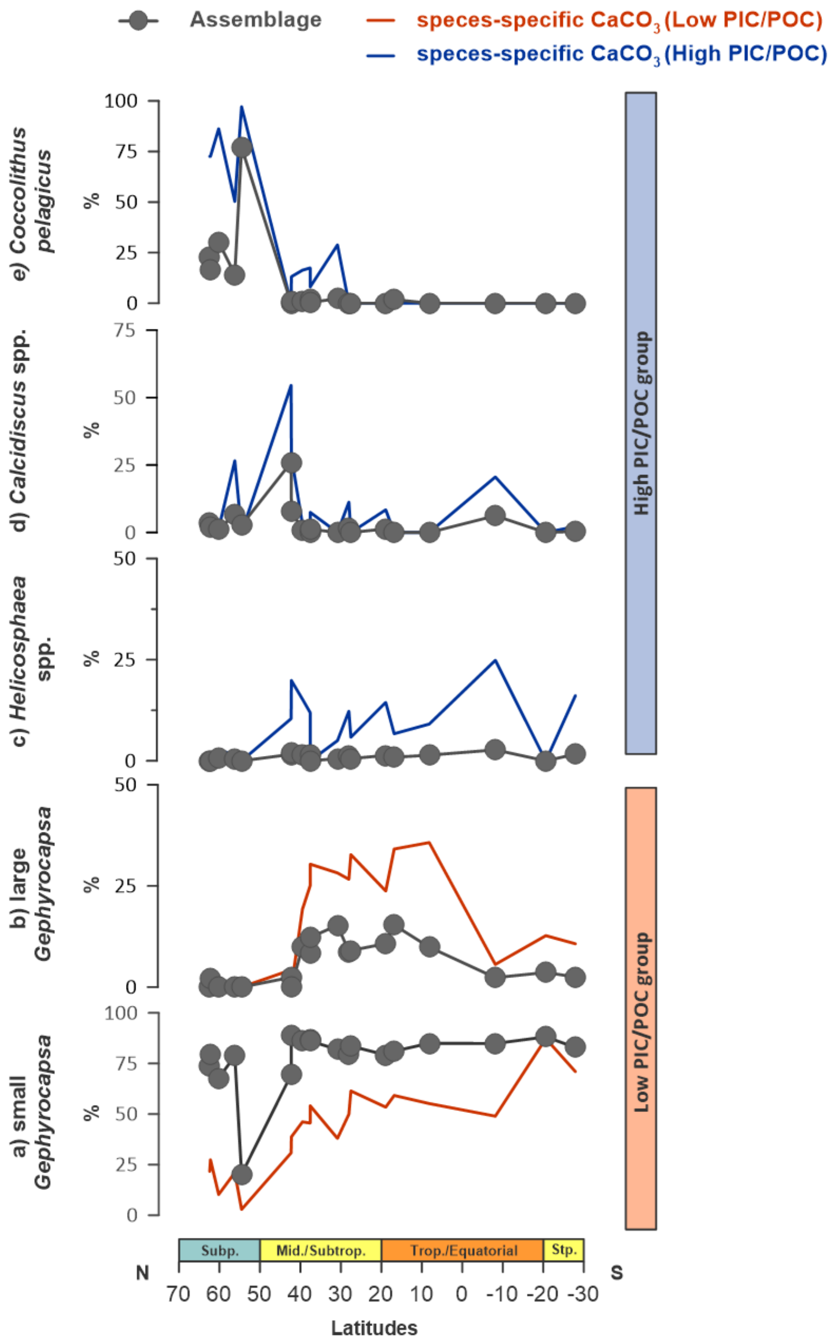

Supplementary Figure 7. Latitudinal variability in the relative representation (%) of the different coccolithophore species within the studied assemblages (grey dots) and their relative contribution to coccolithophore calcite (orange: taxa belonging to the low PIC/POC group; blue: taxa belonging to the high PIC/POC group). **a)** small *Gephyrocapsa*; **b)** large *Gephyrocapsa*; **c)** *Helicosphaera* spp., **d)** *Calcidiscus* spp. and **e)** *C. pelagicus*.

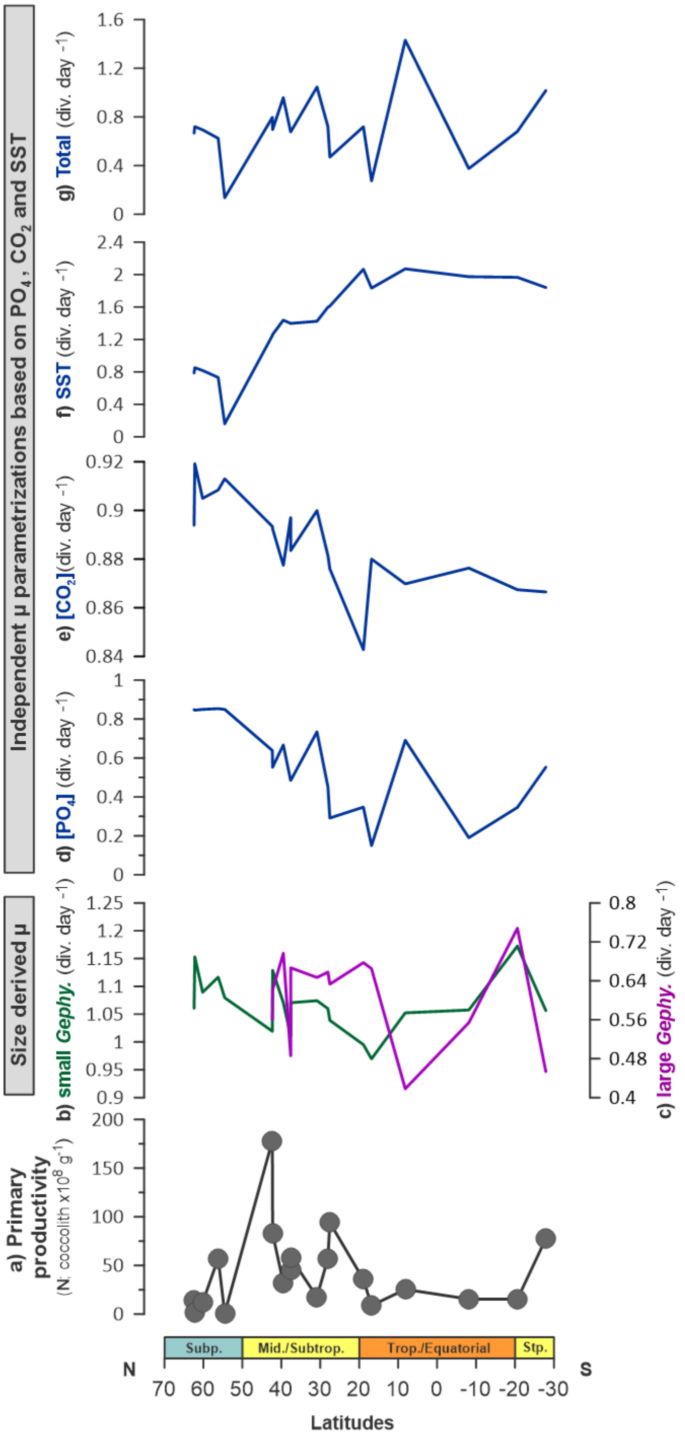

Supplementary Figure 8. Comparison between primary productivity estimates and growth rate (μ) parametrizations. **a)** Primary productivity from the absolute abundance of coccoliths in sediments (N; coccolith x10^8 g^{-1}); Size-based derivation of taxa-specific μ for **b)** small *Gephyrocapsa* and **c)** large *Gephyrocapsa* (low PIC/POC group) with the formula by Zhang *et al.* [6]; **d-f)** Parametrizations of μ based in surface environmental parameters: surface phosphate concentration ([PO_4]; μmol/L), surface CO_2 concentration ([CO_2]; μmol/kg) and Sea Surface Temperatures (SST; °C), calculated with the formula by Krumhardt *et al.* [7]; **g)** μ parametrization with the integrated effect of [PO_4], [CO_2] and SST parameters [7]. Environmental parameter values were extracted from databases GLODAPv2.2023 [8,9,10] and WOA [11,12]. Values are summarized in the Supplementary Table 4 (see Methods in Main Text for calculations).

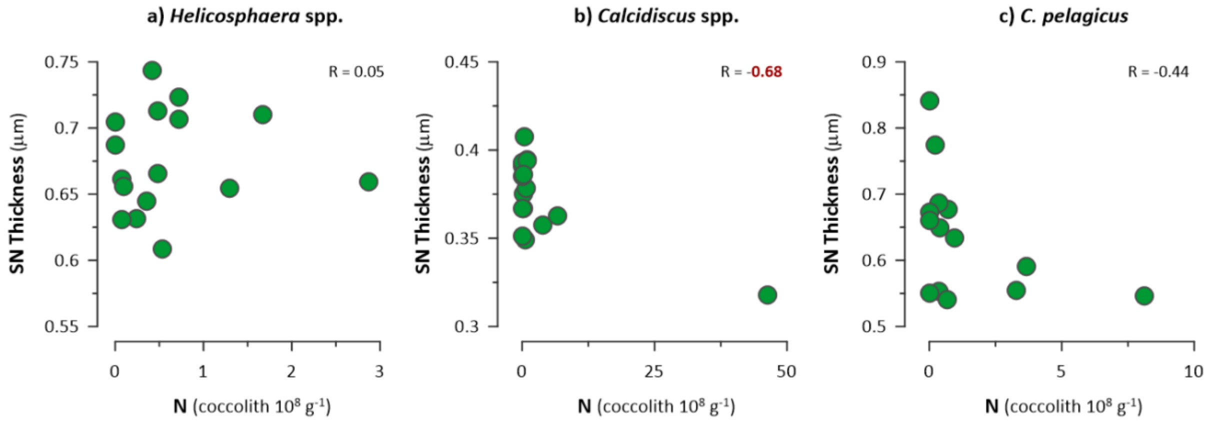

Supplementary Figure 9. Cross correlation relationship between micropaleontological coccolith absolute abundances (N; coccolith x10^8 g^{-1}) and Size Normalized (SN) Thickness (μm) for the high PIC/POC coccolithophore taxa: **a)** *Helicosphaera* spp.; **b)** *Calcidiscus* spp. and **c)** *C. pelagicus*.

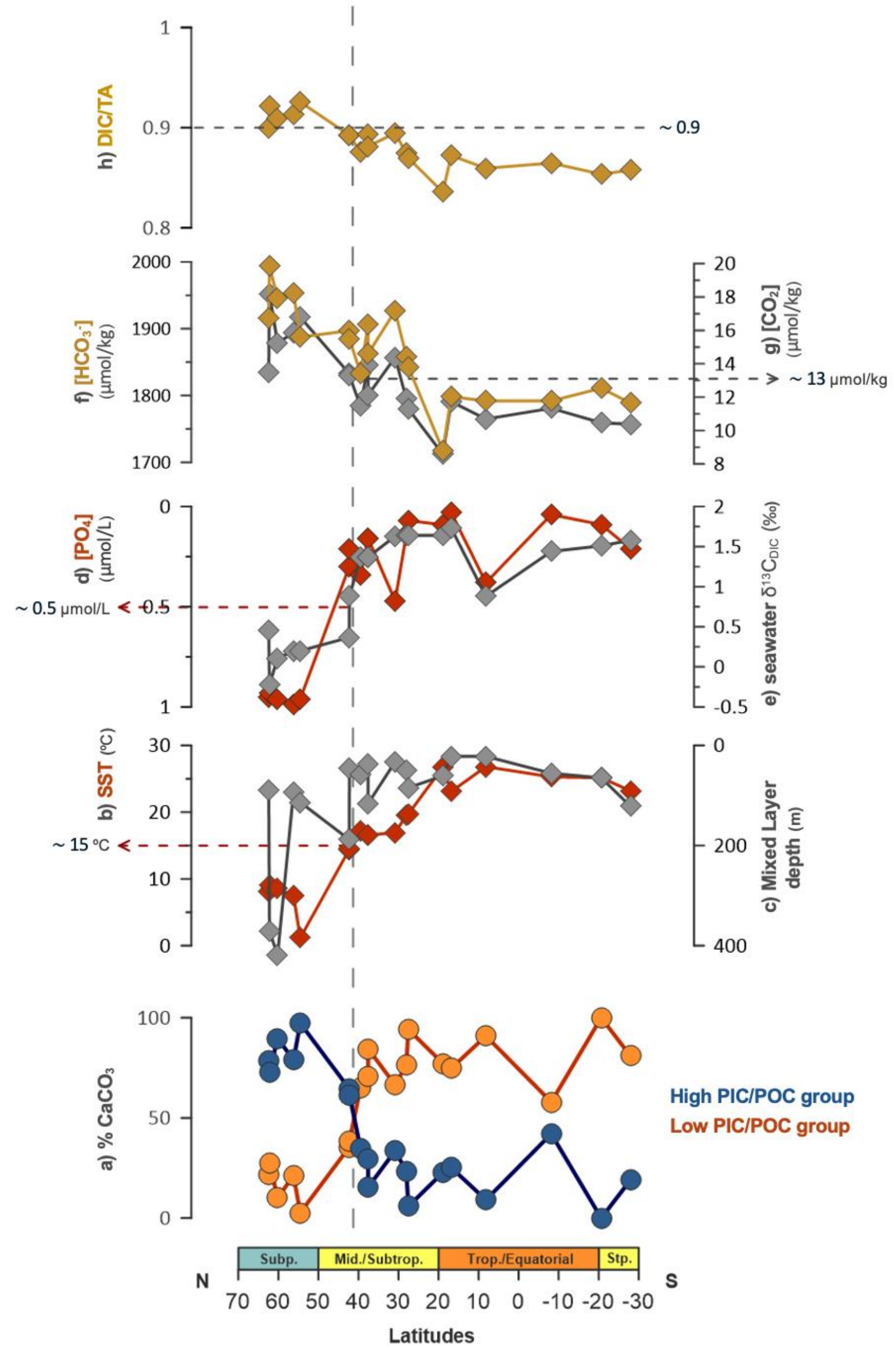

Supplementary Figure 10. Representation of the relative (%) contribution to coccolithophore calcite of **a)** low PIC/POC group (orange) and high PIC/POC group (blue), compared with environmental parameters discussed in the Main Text: **b)** Sea Surface Temperatures (SST; °C); **c)** Mixed layer depth (m); **d)** surface phosphate concentration ([PO_4]; µmol/L); **e)** surface seawater carbon isotopic ratio of DIC ($\delta^{13}C_{DIC}$; ‰); **f)** surface bicarbonate concentration ([HCO_3^-]; µmol/kg); **g)** surface CO_2 concentration ([CO_2]; µmol/kg) and **h)** the ratio of Dissolved Inorganic Carbon to Total Alkalinity (DIC/TA ratio). The bimodal regime, with a boundary at ~40° N, is indicated by a vertical dashed line; values of SST, [PO_4], [CO_2] and DIC/TA ratio at this boundary are indicated by arrows on the corresponding axes. Environmental parameter values were extracted from databases GLODAPv2.2023 [8,9,10] , WOA [11,12] and from Eide *et al.* [13] (see Methods in Main Text for calculations).

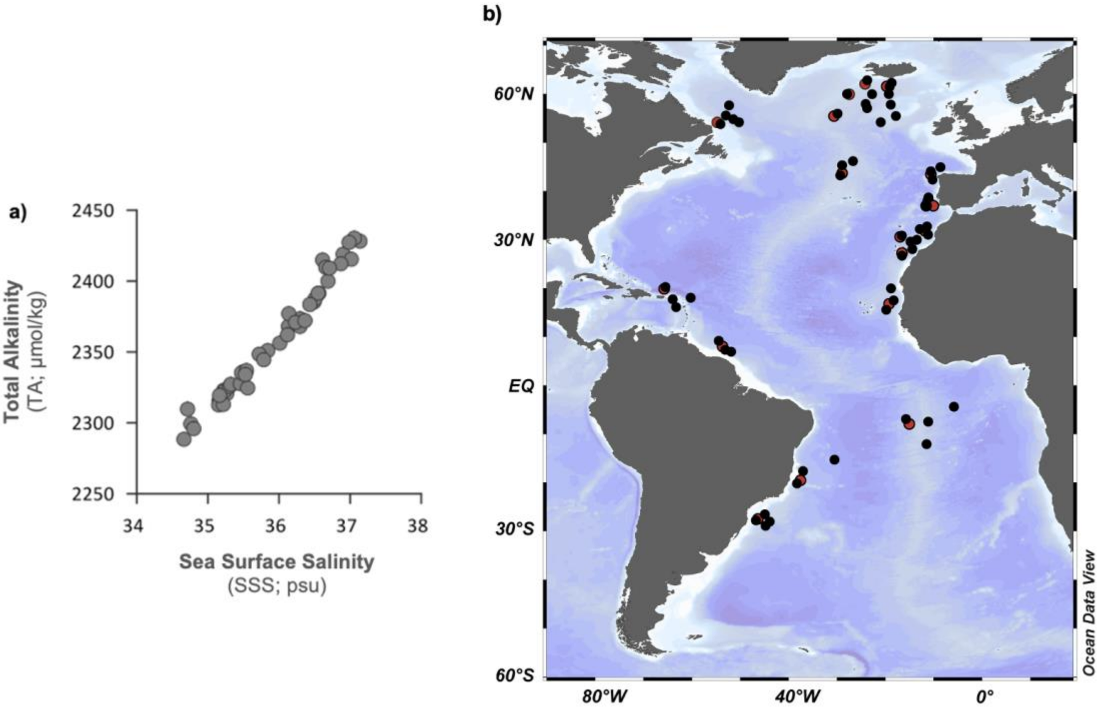

Supplementary Figure 11. a) Correspondence between Sea Surface Salinities (SSS; PSU) and surface Total Alkalinity (TA; µmol/kg) across latitudes. Environmental SSS and TA values were extracted from a high-resolution dataset at a fixed depth of 100 meters from World Ocean Atlas (WOA) [11,12]; **b)** Map showing the latitudes used for representation generated using Ocean Data View [1]. The bathymetry from ETOPO5 [2] is incorporated by ODV for the map representation.

Supplementary Tables

Sample	Expedition	Latitude	Longitude	Water depth (m)
NEAP 1B	*NEAPACC*	62.40	-22.27	1092
RAPID13-9B	*CD159*	62.22	-17.99	1751
NEAP 7B	*NEAPACC*	60.20	-23.40	1997
NEAP 16B	*NEAPACC*	56.21	-27.49	2847
RAPID38-27B	*CD159*	54.53	-52.43	2159
NEAP 20B	*NEAPACC*	42.30	-28.20	2878
GeoB15629-1	*Meteor 84/4*	42.24	-9.62	1885
SHAK09 8M	*JC089*	39.49	-9.35	2323
SHAK06 5M	*JC089*	37.60	-10.10	2445
SHAK12 10B	*JC089*	37.58	-9.35	1611
GeoB6007-1	*M45/5*	30.85	-10.27	899
M24/1_8-1	*M24/1*	28.04	-14.98	1496
GeoB5538-2	*M42/4b*	27.54	-15.12	2537
M35/1_10-2	*M 35/1*	18.93	-64.09	2696
GeoB9501-4	*M65/1*	16.84	-16.73	330
GeoB7011-2	*M49/4*	8.18	-53.68	1205
GeoB5002-1	*M41/2*	-8.14	-14.54	2851
GeoB2130-1	*M23/2*	-20.62	-37.10	2113
GeoB2109-3	*M23/2*	-27.91	-45.87	2513

Supplementary Table 1. Metadata information of samples in this study: Complete sample reference name from repository, oceanographic expedition, latitude, longitude and water depth.

Sample	Core type	Age	Protocol	Age determination / Application
NEAP 1B	Box Core	< 3000	Radiocarbon	Rickaby and Elderfield (1999) / Yu et al. (2007)
RAPID13-9B	Box Core	< 1390	Equivalent to surfacemost RAPID-10-1P	Thornalley et al. (2010) / Yu et al. (2013)
NEAP 7B	Box Core	< 3000	Radiocarbon	Rickaby and Elderfield (1999) / Yu et al. (2007)
NEAP 16B	Box Core	< 3000	Radiocarbon	Rickaby and Elderfield (1999) / Yu et al. (2007)
RAPID38-27B	Box Core	< 1000	Radiocarbon	Yu et al. (2013)
NEAP 20B	Box Core	< 3000	Radiocarbon	Rickaby and Elderfield (1999) / Yu et al. (2007)
GeoB15629-1	Multicorer	late Holocene	Equivalent to surfacemost GeoB15629-2	Petrovic et al. (2019) / This study
SHAK09 8M	Box Core	< 1300	Equivalent to surfacemost MD99-2334K and SHAK06-5K	Skinner et al. (2014); Ausin et al. (2019) / This study
SHAK06 5M	Box Core	< 1300	Equivalent to surfacemost MD99-2334K and SHAK06-5K	Skinner et al. (2014); Ausin et al. (2019) / This study
SHAK12 10B	Box Core	< 1300	Equivalent to surfacemost MD99-2334K and SHAK06-5K	Skinner et al. (2014); Ausin et al. (2019) / This study
GeoB6007-1	Multicorer	modern - late Holocene	Radiocarbon	Eberwein (2007) / Morley (2011)
M24/1_8-1	Giant Box Core	late Holocene	Assumed as simmilar to GeoB6007-1 and GeoB5538-2	This study
GeoB5538-2	Multicorer	late Holocene	Equivalent to surfacemost GeoB4216 and GeoB4241	Henderiks et al. (2002)
M35/1_10-2	Giant Box Core	< 3000	Radiocarbon	Regenberg et al.(2006) / Steph et al. (2009)
GeoB9501-4	Multicorer	modern	$^{210}Pb/^{137}Cs$	Mulitza et al. (2010)
GeoB7011-2	Multicorer	late Holocene	Equivalent to surfacemost MD95-2042	Shakleton et al. (2000) / Govin et al. (2014)
GeoB5002-1	Multicorer	modern - late Holocene	Comparison with surface conditions	Vink et al. (2004)
GeoB2130-1	Multicorer	modern - late Holocene	Comparison with surface conditions	Mulitza et al. (1998)
GeoB2109-3	Multicorer	late Holocene	Isotope stratigraphy; equivalent to surfacemost GeoB2805-1	Dürkoop (1998); Mollenhauer et al. (2006) / Groeneveld and Chessi (2011)

Supplementary Table 2. Summary of age information for the samples analyzed in this study. For each sample: reference name, drilling/core type, estimated sample age, the protocol used for age assignment in previous studies, and the corresponding references. The references cited corresponds to the study in which the age-assignment protocol was applied and from which the assumed age range was derived. Extended information is included in Methods S1.

**Ω Calcite depth**	small *Gephy.*	large *Gephy.*	*Helico.* spp.	*Calcidiscus* spp.	*C. pelagicus*
Size (µm)	-0.3	0.06	0.1	0.17	0.27
SN Thickness (µm)	-0.1	0.15	-0.09	-0.02	-0.18

Supplementary Table 3. Correlation analysis between the values of saturation of calcite at depth ($\Omega_{\text{Calcite depth}}$) and the morphometric parameter of Size (µm) and the calcification index SN Thickness (µm).

Sample	$\delta^{13}C_{DIC}$ (‰)	SST (ºC)	SSS (PSU)	$[PO_4]$ (µmol/L)	Mixed Layer (depth; m)	TA (µmol/kg)	pH	$[CO_2]$ (µmol/kg)	$[HCO_3^-]$ (µmol/kg)	$[CO_3^{2-}]$ (µmol/kg)	DIC (µmol/kg)	$[H^+]$ (µmol/kg)	ΩCa surf.	ΩCa depth
NEAP 1B	0.47	8.18	35.14	0.95	89.75	2323.90	8.17	13.48	1916.15	161.29	2090.80	6.83	3.77	1.65
RAPID13-9B	-0.23	9.01	35.20	0.93	370.75	2324.60	8.04	18.20	1993.71	130.90	2142.65	9.07	3.06	1.86
NEAP 7B	0.11	8.56	35.15	0.96	418.75	2319.60	8.11	15.24	1945.54	147.92	2108.55	7.68	3.46	1.69
NEAP 16B	0.19	7.48	35.06	0.99	93.50	2310.20	8.11	15.87	1952.99	140.92	2109.63	7.74	3.29	1.59
RAPID38-27B	0.20	1.16	33.71	0.96	113.63	2179.10	8.15	16.79	1887.45	113.03	2017.10	7.00	2.67	1.69
NEAP 20B	0.37	14.40	35.92	0.30	188.13	2336.70	8.09	13.41	1897.09	175.34	2085.73	8.08	4.08	1.69
GeoB15629-1	0.88	14.58	35.62	0.21	45.40	2321.30	8.09	13.27	1884.06	174.43	2071.66	8.06	4.07	1.73
SHAK09 8M	1.37	17.13	36.21	0.34	58.40	2335.60	8.12	11.46	1833.42	200.66	2045.45	7.62	4.67	1.73
SHAK06 5M	1.37	16.55	36.11	0.16	37.56	2347.20	8.06	13.94	1906.20	176.46	2096.48	8.79	4.11	1.61
SHAK12 10B	1.37	16.55	36.11	0.16	115.91	2346.30	8.11	12.14	1862.61	193.50	2068.15	7.83	4.50	1.55
GeoB6007-1	1.63	16.94	36.31	0.47	33.00	2366.60	8.04	14.39	1926.49	176.20	2116.97	9.07	4.10	2.23
M24/1_8-1	1.64	19.50	36.72	0.14	50.41	2376.00	8.08	11.90	1858.64	207.68	2078.13	8.26	4.82	1.96
GeoB5538-2	1.64	19.72	36.68	0.07	84.50	2379.10	8.10	11.30	1842.42	215.58	2069.20	7.94	5.01	1.52
M35/1_10-2	1.64	26.74	35.69	0.09	61.00	2392.90	8.13	8.58	1717.46	273.71	1999.69	7.40	6.48	1.41
GeoB9501-4	1.73	23.09	35.83	0.03	22.00	2311.60	8.05	11.73	1798.16	206.37	2016.18	9.01	4.85	1.52
GeoB7011-2	0.88	26.80	34.59	0.38	22.00	2370.80	8.06	10.69	1791.72	235.36	2037.70	8.77	5.62	1.87
GeoB5002-1	1.44	25.27	36.20	0.04	55.83	2341.00	8.04	11.33	1791.69	221.36	2024.31	9.14	5.20	1.30
GeoB2130-1	1.51	25.15	37.11	0.09	65.00	2423.80	8.08	10.47	1810.89	247.71	2069.00	8.39	5.78	1.65
GeoB2109-3	1.58	23.20	36.71	0.21	121.50	2370.60	8.09	10.39	1788.65	234.14	2033.10	8.09	5.46	1.68

Supplementary Table 4. Set of surface environmental parameters used in this study. Data values are extracted from Eide *et al.*[13] and the databases GLODAPv2.2023 [8,9,10] and WOA [11,12]. Details on extraction methods, corrections and calculations are provided in the main text Methods section.

Growth rates μ (div. day^{-1})	small *Gephy.* μ size	large *Gephy.* μ size
**μ [CO_2]**	0.41	-0.15
**μ SST**	-0.34	-0.11
**[PO_4]**	0.43	-0.29

Supplementary Table 5. Correlation analysis between the growth rate parametrizations (μ) in this study.

Supplementary Methods

Supplementary Methods 1. Age information.

A majority of the surface sediment samples used in this study have been previously analyzed in the context of proxy calibration (NEAP 1B, RAPID 13-9B, NEAP 7B, NEAP 16B, RAPID 38-27B, NEAP 20B, M35/1_10-2 and GeoB2130-1) [14-18], evaluated against modern environmental conditions (GeoB5002-1, GeoB2130-1) [19,20] and/or dated using absolute age constraints (GeoB6007-1, GeoB9501-4 and GeoB2109-3) [21-24]. For some samples (GeoB5538-2, GeoB7011-2, and GeoB2109-3), available age determinations, considered in our study, are based on the correspondence with nearby well-dated records reported in previous studies [25-27]. A similar approach is applied here for samples GeoB15629-1, SHAK09, SHAK06, SHAK12, and M24/1_8-1, using reference sites with independently established chronologies [22, 28-30]. In summary, the surface sediment samples analyzed in this study represent relatively modern conditions, with maximum estimated ages falling within the late Holocene. The supplementary Table S2 summarizes the relevant references, methods, and age estimates for all samples.

Supplementary Methods 2. Calculation of calcification indexes: Size Normalized (SN) Thickness, elliptical shape factor (kse) and morphometric PIC/POC.

To evaluate changes in coccolith thickness within each coccolith group analyzed in this study, the values of thickness are normalized by following the calculation for Size Normalized (SN) Thickness protocol, as proposed by O'Dea *et al.* [31]:

$$SN\ Thickness = [(ML - CL) * S] + CT \quad (1)$$

ML = mean coccolith length; CL = length of each individual coccolith in sample; S = slope of the regression between coccolith length and coccolith thickness for all coccoliths in sample; CT = original thickness of each individual coccolith in sample. Values of length correspond to the size of the coccolith major axis.

The shape factor (ks) is a dimensionless estimate of the fraction of a cube of a given side length that is occupied by the volume of a coccolith [32]. In this study, the shape factor is calculated relative to the volume of an ellipsoid defined by the length of the major and minor axes of the coccolith, referred as elliptical shape factor (kse) [33]. This provides a more accurate estimate, as it accounts for the natural elliptical shape of most coccoliths:

$$kse = \frac{v}{\frac{4}{3}\pi\frac{l}{2}(\frac{w}{2})^2} \quad (2)$$

l = length (i.e., size major axis); w = width (i.e., size minor axis).

The inorganic/organic carbon ratio (PIC/POC) integrates the rates of calcification to photosynthesis [34]. For the calculation of this parameter in low PIC/POC taxa (i.e., small and large *Gephyrocapsa*), we use the morphometric measurements of coccolith length and thickness. Following the approach by Bolton *et al.* [35], POC/cell is estimated based on cellular carbon quotas [36], derived from cell radius and surface area calculations from coccolith length. PIC/cell is calculated from PIC/surface area estimates from coccolith thickness, based in the regression for Noelaerhabdaceae coccoliths [37], to which the low PIC/POC taxa belong. This parameter is expressed as pg inorganic carbon / organic carbon (pg C inorg. / org.).

Supplementary Methods 3. Mass calculations

The structure of the coccoliths produced by *Gephyrocapsa* (low PIC/POC group) is composed by radially oriented crystal units (i.e., R units). In contrast, coccoliths produced by the high PIC/POC groups, *Helicosphaera* spp., *Calcidiscus* spp. and *C. pelagicu*s, are variably composed of two distinct crystal unit types: the radially oriented, R Units, and the vertically oriented, V units [38]. The different birefringence properties of these type of units may introduce some bias in the two dimensional morphometric estimates (i.e., volume) of coccoliths composed by mixed crystal unit structures when using a surface-birefringence based method [39]. Although this does not limit our assessment of within-species variability, it may introduce a degree of methodological bias in the absolute mass quantification of the high PIC/POC groups. To better account for this potential influence on mass estimates, we complemented our analysis with the independent protocol developed by Young and Ziveri [32]. To apply this approach, we used the average size measurements, derived from C-Calcita, along with group-specific recommended ks value (*Helicosphaera* spp. = 0.05; *Calcidiscus* spp. = 0.08; *C. pelagicus* = 0.06; *Gephyrocapsa* = 0.05) [32]. Because this method relies on a constant, literature-based, ks value that does not vary among samples, it also carries its own, and potentially larger, sources of uncertainty. All calculations of calcite mass have been performed with both methods and, despite the different uncertainties, the differences in trends and environmental relationships with mass from each method are negligible. This study does not aim to quantify absolute $CaCO_3$ contributions; therefore, these methodological uncertainties do not represent a limitation for our purposes. What is essential is the consistent use of mass values calculated with the same method for both calcifying groups (low and high PIC/POC), ensuring that interdependent relative contributions to coccolithophore $CaCO_3$ remain methodologically coherent. For all figures in the Main Text and Supplements, masses derived from C-calcita have been used.

Supplementary Notes

Supplementary Notes 1. Evaluation of preservation and representativeness of surface sediments

Building on the general good preservation characteristics of the studied settings (namely shallow water depths above the Atlantic Carbonate Compensation Depth and $\Omega_{\text{Calcite depth}}$ states consistently above undersaturation thresholds, Main Text and Table S4), we performed additional assessments to further evaluate the preservation state of the surface sediments and the robustness of the micropaleontological and morphometric signal.

First, the dominance of the small *Gephyrocapsa* group, accounting for over 70% of the total assemblage in our samples (Supplementary Figure 7), strongly supports good preservation in the studied sediments. Dominance of this group (mostly composed by *G. huxleyi*, the formerly termed *Emiliania huxleyi* before Bendif *et al.* [40]) is consistent with well-preserved

surface sediment records across the Atlantic [41,42] and with the composition of modern coccolithophore communities [43], in which this group also predominates. Due to their smaller size and thinner calcite structure, the coccoliths produced by this group are more prone to dissolution; in poorly preserved assemblages, they are often significantly underrepresented [44]. Their clear dominance in our dataset, not only reflects their cosmopolitan distribution and wide ecological adaptability [45], but also serves as indicator of good preservation of assemblage structure.

Additionally, the absence of significant correlations between group-specific morphometric parameters and indexes with the $\Omega_{\text{Calcite depth}}$ (Supplementary Table 3) indicates that poor preservation/dissolution at the seabed does not exert a dominant control on those morphometric profiles and indexes. Ranges of size, mass, thickness, and SN Thickness in this study (Supplementary Figure 4) are consistent along groups with reference data from well-preserved records analyzed with the application of the same image analysis methodology [33,35,46-48]. Sample selection at depths below the CCD, with a limited range of maximum depth and $\Omega_{\text{Calcite depth}}$ variability and positioned well above the level of saturation of calcite (Supplementary Figure 2 and Table 4), appears to be an effective strategy to avoid poor preservation conditions affecting coccolith morphometries in more dissolution prone settings with a wider range of variability in depth and saturation states [49].

The percentage of % $CaCO_3$ in sediments along the studied regions remains stable, ranging between 40-60 % along most of the record (Supplementary Figure 3), with slight deviations from that range in full agreement with surface production patterns. Regions with $CaCO_3$ values over 60 % are consistent with enhanced productivity values in computational estimates of yearly summed net Atlantic primary productivity [50,51]. Low % $CaCO_3$ below 40% at the Labrador Sea region agrees with overally reduced primary productivity and coccolithophore representation at this region in previous research [52]. A sample exhibiting low % $CaCO_3$ at the western boundary tropical region is located outside the accumulation rate maxima associated with Amazon river discharge (Supplementary Figure 3). While providing terrigenous supply (i.e., clay), the influence of Amazon plume is known to jointly lead surface ocean fertilization, resulting in high coccolithophore primary productivity at this region (see discussion in Gonzalez-Lanchas *et al.* [53]). As such, reduced % $CaCO_3$ in that regional domain could be considered to dominantly relate to reduced surface fertilization and primary productivity.

Supplementary Notes 2. Coccolith morphometries

A positive relationship is always observed between coccolith size and mass measurements for each of the studied taxa (Supplementary Figure 4). This correspondence builds on previous research targeting specimens belonging to both the low and high PIC/POC groups, in both nannofossil research [33,47] and culture-based experiments [54], and indicates a dominant control of size variability over coccolith mass change. A correspondence between coccolith size variability and thickness change is similarly observed for all the coccolithophore groups (Fig. S4). This relationship confirms the existence of a geometrically natural allometric control of size variability over thickness change [31], that is common to all the groups considered in this study.

After calculating Size Normalized (SN) Thickness, we observe a decoupling between size variability and thickness change (Supplementary Figure 4), that confirms the normalization of allometric thickness change to size variability with this calculation [31].

Supplementary Notes 3. Latitudinal trends in assemblages and morphometry.

The absolute abundances of the total coccolithophore assemblages (N Total) exhibit maximum values in the mid-latitudes to subtropical regions, located between the 50° to 20°N and south the 20°S (Figure 2), in agreement with both enhanced sedimentary % $CaCO_3$ values (Supplementary Figure 3) and enhanced primary productivity in the computational estimates of yearly summed net Atlantic values [50,51]. Together with the assessed non-dominant effect of dissolution (Supplementary Text 1), this correspondence supports the application of N as a qualitative indicator of the variability in surface primary productivity across the Atlantic, agreeing with classic application of this proxy in micropaleontological studies [55-59]. The correspondence of production and distribution patterns of groups in this research with existing Holocene to modern studies [42,43,59-60] provides fidelity on the representativeness of our sedimentary data and the consideration of profiles as a mean for discussion of coccolithophore physiology. Below we separately describe the specific results from the two studied groups.

The low PIC/POC group (primarily small *Gephyrocapsa*) is dominant across the studied latitudes with the exception of a sample at 55° N. This biogeographic distribution agrees with exiting characterizations from both surface sediments and water samples covering the Holocene to modern period in the Atlantic [42,43,61]. In agreement with the computational primary production estimates [50,51], the primary productivity profile (i.e., N) of the dominant small *Gephyrocapsa* exhibit well the general trend of enhanced production conditions in the mid-latitudes to subtropical regions, with reduced production along the tropics and equator (Figures 2 and 3). The primary productivity profile of the large *Gephyrocapsa* group exhibit a highly similar trend, regardless the representation of this group in assemblages is lower (Figure 2 and Supplementary Figure 7). Notably, the primary productivity profiles of each small and large *Gephyrocapsa* group show a tight correspondence with their respective calcification intensity patterns along latitudes (i.e., correspondence between N, SN Thickness and PIC/POC; Figure 2).

The coccolithophore groups categorized as high PIC/POC producers, *Helicosphaera* spp., *Calcidiscus* spp. and *C. pelagicus* have a general minor representation over assemblages. Summed relative contributions are below 10 % along the record, with the exception of the mid to high latitude sector 40-55°N in which an enhanced representation of *Calcidiscus* spp. and *C. pelagicus* is recorded (Figure 2 and Supplementary Figure 7). The maximum primary productivity values (i.e., N) of these three groups are restricted to latitudes north of 40°N in this record (Figure 2). In particular, enhanced primary productivity of *C. pelagicus* are found in subpolar environments between, 55° to 65°N, while elevated primary productivity of *Calcidiscus* spp. and *Helicosphaera* spp., are registered between 35° to 45°N (Figure 2). This biogeographic distribution agrees with exiting characterizations from both surface sediments and water samples covering the Holocene to modern period in the Atlantic [42,43,59-63].

There is a negative correspondence between the primary productivity profiles of the high PIC/POC taxa *Calcidiscus* spp. and *C. pelagicus* with their SN Thickness profiles (Figure 2 and Supplementary Figure 9).

Supplementary Notes 4. Coccolithophore sedimentary calcite concentration and group-specific contribution

Following the confirmation that N values serve as appropriate qualitative indicators of primary productivity and that coccolith morphometries are non-dominantly affected by dissolution, we interpret the coccolithophore sedimentary calcite concentrations as a reasonable approximation of coccolithophore calcite production (Figure 2 and Supplementary Figure 10). Still, our analysis does not involve evaluation of absolute quantifications, but rather focuses on

latitudinal trends and the relative contributions between the coccolithophore groups considered and separately categorized as low and high PIC/POC producers.

Coccolithophore calcite production follows the same trend as total primary productivity, with enhanced values at the mid-latitudes to subtropical regions, at latitudes between 50 to 20°N and below the 20°S. The high PIC/POC group is the dominant contributor at latitudes above ~40°N in this record, with an increased role (>75 % contribution) towards the north. The low PIC/POC group is the dominant contributor at latitudes below ~40°N (>75 % contribution; Figure 2 and Supplementary Figure 10). At those regions enhanced representation of the high PIC/POC group and reduced representation of the low PIC/POC group, reaching both comparable values, is just observed at a discrete sample at 8°S.

There is a latitudinal correspondence between the profile of contribution of the low PIC/POC group to coccolithophore calcite production and the calcification intensity of the components within this group. Highly calcified and more productive specimens characterize the regions of enhanced contribution of this group to coccolithophore calcite. For its part, the profile of contribution of the high PIC/POC group to coccolithophore calcite production along latitudes does not show a latitudinal correspondence with the profiles of calcification intensity of the components within this group. Less calcified specimens, under conditions of enhanced productivity, characterize the regions of enhanced contribution of this group to coccolithophore calcite (Figure 2).

Supplementary References

1 Schlitzer, R. Ocean Data View, odv.awi.de. https://odv.awi.de/ (2022)

2 National Geophysical Data Center, 1993. 5-minute Gridded Global Relief Data (ETOPO5). National Geophysical Data Center, NOAA. doi:10.7289/V5D798BF

3 Schlitzer, Reiner and Sebastian Mieruch-Schnülle, webODV Explore, https://explore.webodv.awi.de, (2026)

4 WOCE Data Products Committee (2002). NODC Standard Product: World Ocean Circulation Experiment (WOCE) Global Data Resource (GDR), versions 1-3, on CD-ROM and DVD. NOAA National Centers for Environmental Information. Dataset. https://www.ncei.noaa.gov/archive/accession/NODC-WOCE-GDR. Accessed March 2026

5 Dittert, Nicolas; Bakker, Dorothee C E; Bendtsen, Jorgen; Corrin, Lydie; Gehlen, Marion; Heinze, Christoph; Maier-Reimer, Ernst; Michalopoulos, Panagiotis; Soetaert, Karline; Tol, Richard S J (2005): Integrated Data Sets of the EU FP5 Research Project ORFOIS: Origin and fate of biogenic particle fluxes in the ocean and their interactions with atmospheric CO2 concentrations as well as the marine sediment [dataset]. World Data Center for Marine Environmental Sciences, PANGAEA, https://doi.org/10.1594/PANGAEA.760906

6 Zhang, Y. G., Henderiks, J. & Liu, X. Refining the alkenone-pCO2 method II: Towards resolving the physiological parameter 'b'. *Geochimica et Cosmochimica Acta* 281, 118-134 (2020).

7 Krumhardt, K. M., Lovenduski, N. S., Iglesias-Rodriguez, M. D. & Kleypas, J. A. Coccolithophore growth and calcification in a changing ocean. *Progress in oceanography* 159, 276-295 (2017).

8 Olsen, A. et al. The Global Ocean Data Analysis Project version 2 (GLODAPv2) – an internally consistent data product for the world ocean. *Earth Syst. Sci. Data* **8**, 297-323 (2016).

9 Key, R. M. et al. Global ocean data analysis project, version 2 (GLODAPv2). Ornl/Cdiac-162, Ndp-093 (2015).

10 Lauvset, S. K. et al. Global Ocean Data Analysis Project version 2.2023 (GLODAPv2. 2023). (2023).
11 Locarnini, M. et al. World ocean atlas 2018, volume 1: Temperature. (2018).
12 Garcia, H. E. et al. World Ocean Atlas 2018, volume 4: Dissolved inorganic nutrients (phosphate, nitrate and nitrate+ nitrite, silicate). (2019).
13 Eide, M., Olsen, A., Ninnemann, U. S. & Johannessen, T. A global ocean climatology of preindustrial and modern ocean δ13C. *Global Biogeochemical Cycles* **31**, 515-534 (2017).
14 Yu, J., Elderfield, H. & Hönisch, B. B/Ca in planktonic foraminifera as a proxy for surface seawater pH. *Paleoceanography* **22** (2007).
15 Yu, J., Thornalley, D. J., Rae, J. W. & McCave, N. I. Calibration and application of B/Ca, Cd/Ca, and δ11B in Neogloboquadrina pachyderma (sinistral) to constrain CO2 uptake in the subpolar North Atlantic during the last deglaciation. *Paleoceanography* **28**, 237-252 (2013).
16 Steph, S., Regenberg, M., Tiedemann, R., Mulitza, S. & Nürnberg, D. Stable isotopes of planktonic foraminifera from tropical Atlantic/Caribbean core-tops: Implications for reconstructing upper ocean stratification. *Marine Micropaleontology* **71**, 1-19 (2009).
17 Kim, J.-H. et al. New indices and calibrations derived from the distribution of crenarchaeal isoprenoid tetraether lipids: Implications for past sea surface temperature reconstructions. *Geochimica et Cosmochimica Acta* **74**, 4639-4654 (2010).
18 Rickaby, R. & Elderfield, H. Planktonic foraminiferal Cd/Ca: paleonutrients or paleotemperature? *Paleoceanography* **14**, 293-303 (1999).
19 Mulitza, S., Wolff, T., Pätzold, J., Hale, W. & Wefer, G. Temperature sensitivity of planktic foraminifera and its influence on the oxygen isotope record. *Marine Micropaleontology* **33**, 223-240 (1998).
20 Vink, A. et al. Coccolithophorid and dinoflagellate synecology in the South and Equatorial Atlantic: improving the paleoecological significance of phytoplanktonic microfossils. In *The South Atlantic in the Late Quaternary*, 101-120 (Springer, 2004).
21 Dürkoop, A. Der Brasil-Strom im Spätquartär: Rekonstruktion der oberflächennahen Hydrographie während der letzten 400 000 Jahre. PhD thesis, Universität Bremen (1998).
22 Eberwein, A. Holocene and last glacial maximum (paleo-) productivity off Morocco: evidence from benthic foraminifera and stable carbon isotopes. (Paläo-) Produktivität im Holozän und Letzten Glazialen Maximum vor Marokko aus benthischen Foraminiferen und stabilen Kohlenstoffisotopen. *Berichte zur Polar-und Meeresforschung (Reports on Polar and Marine Research)* **548** (2007).
23 Mollenhauer, G., McManus, J. F., Benthien, A., Müller, P. J. & Eglinton, T. I. Rapid lateral particle transport in the Argentine Basin: molecular 14C and 230Thxs evidence. *Deep Sea Research Part I: Oceanographic Research Papers* **53**, 1224-1243 (2006).
24 Mulitza, S. et al. Increase in African dust flux at the onset of commercial agriculture in the Sahel region. *Nature* **466**, 226-228 (2010).
25 Govin, A. et al. Terrigenous input off northern South America driven by changes in Amazonian climate and the North Brazil Current retroflection during the last 250 ka. *Climate of the Past* **10**, 843-862 (2014).
26 Groeneveld, J. & Chiessi, C. M. Mg/Ca of Globorotalia inflata as a recorder of permanent thermocline temperatures in the South Atlantic. *Paleoceanography* **26** (2011).
27 Henderiks, J. et al. Glacial–interglacial variability of particle accumulation in the Canary Basin: a time-slice approach. *Deep Sea Research Part II: Topical Studies in Oceanography* **49**, 3675-3705 (2002).

28 Petrovic, A. et al. Post-LGM upward shift of the Mediterranean Outflow Water recorded in a contourite drift off NW Spain. *Marine Geology* **407**, 334-349 (2019).
29 Skinner, L. C., Waelbroeck, C., Scrivner, A. E. & Fallon, S. J. Radiocarbon evidence for alternating northern and southern sources of ventilation of the deep Atlantic carbon pool during the last deglaciation. *Proceedings of the National Academy of Sciences* **111**, 5480-5484 (2014).
30 Ausín, B. et al. Radiocarbon age offsets between two surface dwelling planktonic foraminifera species during abrupt climate events in the SW Iberian margin. *Paleoceanography and Paleoclimatology* **34**, 63-78 (2019).
31 O'Dea, S. A. et al. Coccolithophore calcification response to past ocean acidification and climate change. *Nature communications* **5**, 5363 (2014).
32 Young, J. R. & Ziveri, P. Calculation of coccolith volume and it use in calibration of carbonate flux estimates. *Deep sea research Part II: Topical studies in oceanography* **47**, 1679-1700 (2000).
33 Guitián, J., Fuertes, M. Á., Flores, J.-A., Hernández-Almeida, I. & Stoll, H. Variation in calcification of Reticulofenestra coccoliths over the Oligocene–Early Miocene. *Biogeosciences* **19**, 5007-5019 (2022).
34 Rost, B. & Riebesell, U. Coccolithophores and the biological pump: responses to environmental changes. In *Coccolithophores: from molecular processes to global impact*, 99-125 (Springer, 2004).
35 Bolton, C. T. et al. Decrease in coccolithophore calcification and CO2 since the middle Miocene. *Nature communications* **7**, 10284 (2016).
36 Popp, B. N. et al. Effect of phytoplankton cell geometry on carbon isotopic fractionation. *Geochimica et cosmochimica acta* **62**, 69-77 (1998).
37 González-Lemos, S., Guitián, J., Fuertes, M.-Á., Flores, J.-A. & Stoll, H. M. An empirical method for absolute calibration of coccolith thickness. *Biogeosciences* **15**, 1079-1091 (2018).
38 Young, J. R., Davis, S. A., Bown, P. R. & Mann, S. Coccolith Ultrastructure and Biomineralisation. *Journal of Structural Biology* **126**, 195-215 (1999).
39 Fuertes, M.-Á., Flores, J.-A. & Sierro, F. J. The use of circularly polarized light for biometry, identification and estimation of mass of coccoliths. *Marine Micropaleontology* **113**, 44-55 (2014).
40 Bendif, E. M. et al. Rapid diversification underlying the global dominance of a cosmopolitan phytoplankton. *The ISME Journal* **17**, 630-640 (2023).
41 Poulton, A. J., Adey, T. R., Balch, W. M. & Holligan, P. M. Relating coccolithophore calcification rates to phytoplankton community dynamics: Regional differences and implications for carbon export. *Deep Sea Research Part II: Topical Studies in Oceanography* **54**, 538-557 (2007).
42 Ziveri, P., Baumann, K.-H., Böckel, B., Bollmann, J. & Young, J. R. Biogeography of selected Holocene coccoliths in the Atlantic Ocean. In *Coccolithophores: From Molecular processes to global impact*, 403-428 (Springer, 2004).
43 De Vries, J. et al. Haplo-diplontic life cycle expands coccolithophore niche. *Biogeosciences* **18**, 1161-1184 (2021).
44 Saavedra-Pellitero, M., Flores, J.-A., Baumann, K.-H. & Sierro, F.-J. Coccolith distribution patterns in surface sediments of Equatorial and Southeastern Pacific Ocean. *Geobios* **43**, 131-149 (2010).
45 Winter, A. Biogeography of living coccolitho-hores in ocean waters. In: Coccolithophores, 161-177 (Cambridge University Press, 1994).

46 Rigual-Hernández, A. et al. Limited variability in the phytoplankton Emiliania huxleyi since the pre-industrial era in the Subantarctic Southern Ocean. *Anthropocene* **31**, 100254 (2020).
47 González-Lanchas, A. et al. Globally enhanced calcification across the coccolithophore *Gephyrocapsa* complex during the mid-Brunhes interval. *Quaternary Science Reviews* **321**, 108375 (2023).
48 Saavedra-Pellitero, M. et al. Calcification and latitudinal distribution of extant coccolithophores across the Drake Passage during late austral summer 2016. *Biogeosciences* **16**, 3679-3702 (2019).
49 Gerotto, A. et al. Fossil coccolith morphological attributes as a new proxy for deep ocean carbonate chemistry. *Biogeosciences* **20**, 1725-1739 (2023).
50 Smyth, T. et al. Determining Atlantic Ocean province contrasts and variations. *Progress in Oceanography* **158**, 19-40 (2017).
51 Tilstone, G., Smyth, T., Poulton, A. & Hutson, R. Measured and remotely sensed estimates of primary production in the Atlantic Ocean from 1998 to 2005. *Deep Sea Research Part II: Topical Studies in Oceanography* **56**, 918-930 (2009).
52 Marshall, N. R. et al. Biogenic carbonate fluxes and preservation in the northwestern Labrador Sea since the Last Glacial Maximum. *Palaeogeography, Palaeoclimatology, Palaeoecology* **576**, 110498 (2021).
53 González-Lanchas, A. et al. Carbon isotopic fractionation of alkenones and Gephyrocapsa coccoliths over the late Quaternary (Marine Isotope Stages 12–9) glacial-interglacial vycles at the western tropical Atlantic. *Paleoceanography and Paleoclimatology* **36**, e2020PA004175 (2021).
54 Yang, M. et al. Opto-Electrochemical Dissolution Reveals Coccolith Calcium Carbonate Content. *Angewandte Chemie International Edition* **60**, 20999-21006 (2021).
55 González-Lanchas, A. et al. A new perspective of the Alboran Upwelling System reconstruction during the Marine Isotope Stage 11: A high-resolution coccolithophore record. *Quaternary Science Reviews* 245, 106520 (2020).
56 Saavedra-Pellitero, M., Flores, J., Baumann, K.-H., Boeckel, B. & Sierro, F. Comparison of different preparation and analysis techniques for quantitative coccolith studies focusing on biogeographic patterns of species. *Micropaleontology*, **57**, 139-161 (2011).
57 Boeckel, B., Baumann, K.-H., Henrich, R. & Kinkel, H. Coccolith distribution patterns in South Atlantic and Southern Ocean surface sediments in relation to environmental gradients. *Deep Sea Research Part I: Oceanographic Research Papers* **53**, 1073-1099 (2006).
58 Ziveri, P., Rutten, A., De Lange, G., Thomson, J. & Corselli, C. Present-day coccolith fluxes recorded in central eastern Mediterranean sediment traps and surface sediments. *Palaeogeography, Palaeoclimatology, Palaeoecology* **158**, 175-195 (2000).
59 Guerreiro, C., de Stigter, H., Cachão, M., Oliveira, A. & Rodrigues, A. Coccoliths from recent sediments of the central Portuguese margin: Taphonomical and ecological inferences. *Marine Micropaleontology* **114**, 55-68 (2015).
60 Baumann, K.-H., Saavedra-Pellitero, M., Böckel, B. & Ott, C. Morphometry, biogeography and ecology of Calcidiscus and Umbilicosphaera in the South Atlantic. *Revue de Micropaléontologie* **59**, 239-251 (2016).
61 McIntyre, A., & Bé, A. W. Modern coccolithophoridae of the Atlantic Ocean—I. Placoliths and cyrtoliths. In *Deep Sea Research and Oceanographic Abstracts*, 14, 561-597 (Elsevier, 1967).

62 Samtleben, C. & Schröder, A. Living coccolithophore communities in the Norwegian-Greenland Sea and their record in sediments. *Marine Micropaleontology* **19**, 333-354 (1992).
63 Samtleben, C. et al. Plankton in the Norwegian-Greenland Sea: from living communities to sediment assemblages—an actualistic approach. *Geologische Rundschau* **84**, 108-136 (1995).
