# Supplementary material for: Atlantic sediments reveal interacting environmental and physiological controls on coccolithophore calcite production
Source: Nat Commun. 2026 May 28;17:4722. doi: 10.1038/s41467-026-73162-5 (PMC13219798; doi:10.1038/s41467-026-73162-5)
Supplement: Supplementary file 2 — Description of Additional Supplementary Files [file 41467_2026_73162_MOESM2_ESM.pdf]

Description of Additional Supplementary Files

Supplementary Data 1: Micropaleontological data, including absolute and relative abundances of each group. Average values and standard deviations of morphometric parameters, size, mass, and thickness. Average values and standard deviations of calcification indexes SN Thickness, kse and morphometric PIC/POC. Calculations of group-specific contributions to absolute and relative coccolithophore calcite. Parameterizations of growth rate based on morphometric parameters and environmental data
